# UniPrimer: A Web-Based Primer Design Tool for Comparative Analyses of Primate Genomes

**DOI:** 10.1155/2012/520732

**Published:** 2012-05-27

**Authors:** Nomin Batnyam, Jimin Lee, Jungnam Lee, Seung Bok Hong, Sejong Oh, Kyudong Han

**Affiliations:** ^1^Department of Nanobiomedical Science and WCU Research Center, Dankook University, Cheonan 330-714, Republic of Korea; ^2^Department of Clinical Laboratory Science, Juseong University, Cheongwon 363-794, Republic of Korea

## Abstract

Whole genome sequences of various primates have been released due to advanced DNA-sequencing technology. A combination of computational data mining and the polymerase chain reaction (PCR) assay to validate the data is an excellent method for conducting comparative genomics. Thus, designing primers for PCR is an essential procedure for a comparative analysis of primate genomes. Here, we developed and introduced UniPrimer for use in those studies. UniPrimer is a web-based tool that designs PCR- and DNA-sequencing primers. It compares the sequences from six different primates (human, chimpanzee, gorilla, orangutan, gibbon, and rhesus macaque) and designs primers on the conserved region across species. UniPrimer is linked to RepeatMasker, Primer3Plus, and OligoCalc softwares to produce primers with high accuracy and UCSC *In-Silico* PCR to confirm whether the designed primers work. To test the performance of UniPrimer, we designed primers on sample sequences using UniPrimer and manually designed primers for the same sequences. The comparison of the two processes showed that UniPrimer was more effective than manual work in terms of saving time and reducing errors.

## 1. Introduction

The field of comparative genomics has emerged as a result of several whole-genome sequencing projects. At present, six primate whole-genome sequences (human, chimpanzee, gorilla, orangutan, gibbon, and rhesus macaque) are available at the UCSC genome browser (http://www.genome.ucsc.edu/) [1–4]. Based on these data sets, several insertions/deletions (INDELs) and coy number variations (CNVs) have been studied by comparing primate genome sequences [5–10]. However, the computational data analysis output should be experimentally verified using the polymerase chain reaction (PCR), quantitative PCR, comparative genome hybridization array, or single nucleotide polymorphism genotyping array. Among the wet-bench methods, PCR is the most popular and easily accessible skill in molecular biology. Primer selection is very important in PCR-based systems because a specific pair of primers should amplify only a single target from a whole genome. In other words, the properties of the primers determine the specificity of PCR.

Several web-based tools for primer design such as Primer3 [11], Primer3Plus [12], PDA [13], PRIMO [14], and PrimeArray [15] have been developed and upgraded. Along with these software, OligoCalc [16] and Oligo Analysis tools (http://www.operon.com/tools/oligo-analysis-tool.aspx/) are available to calculate the molecular weight, GC content, melting temperature, intermolecular self-hybridization, and intramolecular hairpin loop formation of oligomers or primers. These web-accessible engines are particularly useful for manually selecting PCR primers and optimizing the PCR assay. Here, we introduce a novel web-based primer design tool, UniPrimer, which compares multiple primate sequences and designs primers for the conserved sequences. Before designing primers, the UniPrimer is linked to the repetitive DNA annotation utility RepeatMasker (http://www.repeatmasker.org/cgi-bin/WEBRepeatMasker/) to eliminate the building of any candidate primers containing repetitive elements. The candidate primers are also linked to OligoCalc for users to easily and rapidly access the properties of the designed primers. “UniPrimer” unites all the necessary algorithms and applications needed for designing good quality primers. Users are able to save considerate amount of time and energy that, otherwise, would have been spared on finding each of these web tools separately, submitting sequence over and over again if several primate genomes are compared and validating primers manually one by one. UniPrimer is an easy-to-use tool at hand that combines preeminent primer designing algorithms available on the internet so far and it is accessible at http://biosw.dankook.ac.kr/UniPrimer/.

## 2. Materials and Methods

### 2.1. Sources

UniPrimer incorporates the search results of popular web-based tools and software to produce output. The following is a list of programs and their brief introductions that we utilized for our study.

**BLAT (see [17])**

It is a popular and one of the most powerful homology search tools used to look up the location of a sequence in the genome or determine the exon structure of an mRNA. It is designed to quickly find DNA sequences of 92% and higher similarity with lengths of 40 bases or more. It also searches protein sequences of 80% and greater similarity. BLAT's speed and sensitivity surpass other tools of its kind; this algorithm is much faster and more accurate.

**RepeatMasker**

(http://www.repeatmasker.org/cgi-bin/WEBRepeatMasker/) It is a program that screens DNA sequences for repetitive elements and low complexity DNA sequences and delivers a detailed annotation of the repeats that are present in the query sequences. Currently, it is more popular than other similar programs and summarizes repetitive elements found in the primate genomic DNA sequences.

**Primer3Plus (see [12])**

It is a web interface for Primer3. It is a program for designing PCR primers, as well as hybridization of oligomers and sequencing primers. While designing primers, it takes into consideration many criteria, such as PCR product size, oligonucleotide melting temperature, and GC content and all these criteria are user specifiable. As a result, the user can get as accurate primer design as possible.

**OligoCalc (see [16])**

It is a web-based oligonucleotide properties calculator that computes single or double-stranded DNA and RNA properties, including molecular weight, solution concentration, melting temperature, estimated absorbance coefficients, self-complementarity, and hairpin loop formation.

**UCSC *In-Silico* PCR**

(http://genome.csdb.cn/cgi-bin/hgPcr/) searches a sequence database with a pair of PCR primers. It is fast in performance as indexing strategy is used for search.

### 2.2. Development Environment

Table 1 shows the development environment for UniPrimer. It was developed on Java server pages based on Apache Tomcat 6.0 and the user interface was written on HTML and jQuery. It is well compatible with recent versions of Mozilla, Safari, and Chrome browsers and is accessible from any computer that has access to the internet.

### 2.3. Work Flowchart of UniPrimer (see Figure 1)

**User Interface**

The first column depicts the user interface and options followed by columns of processing steps. Only first column contents are visible to user.

**BLAT (see [17])**

**Figure 1:**
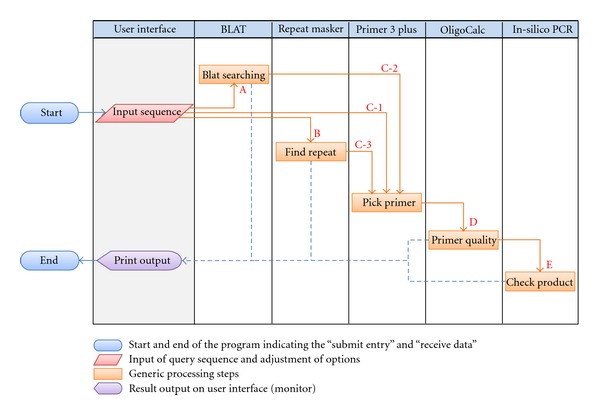
UniPrimer work flow chart. The flowchart represents an overall process for UniPrimer. The sequential steps are denoted as red alphabetical order and numbers. Start and End symbols, pictured as circles, indicate the “submit entry” and “receive product”, respectively.

When user submits a sequence, UniPrimer processes BLAT (step A in Figure 1), where it searches for sequences of high similarity to the user's input. Percent identity can be either a user defined value or a default (>93% depending on the divergence of humans).

**RepeatMasker**

[http://www.repeatmasker.org/cgi-bin/WEBRepeatMasker/] In the next step (step B in Figure 1), the program identifies repetitive elements in query sequences and returns their detailed annotation.

**Primer3Plus (see [12])**

Primers can be designed on any region of query sequence (step C-1 in Figure 1). However, if user wants to get better PCR result, he should take into account the outputs of BLAT (step C-2 in Figure 1) and RepeatMasker (step C-3 in Figure 1).

**OligoCalc (see [16])**

UniPrimer is able to check the self-complementarity of candidate primers using OligoCalc (step D in Figure 1). If the candidate primers do not contain a potential hairpin formation, 3′ complementary, and all potential self-annealing sites, it returns “NONE”. Thus, the user is able to choose primers with good quality for the PCR assay.

**UCSC *In-Silico* PCR**

[http://genome.csdb.cn/cgi-bin/hgPcr/] a final step (step E in Figure 1)is that UniPrimer searches for a sequence with a pair of PCR primers using UCSC *In-Silico* PCR. When successful, it returns a primer pair with the sequence lying between them. A more detailed review of the above steps is included in UniPrimer interface section.

## 3. Results and Discussion

### 3.1. UniPrimer Interface

#### 3.1.1. Input

**Figure 2:**
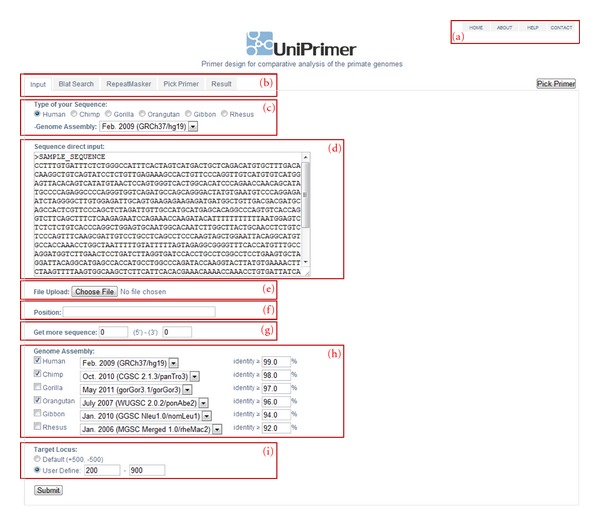
UniPrimer input interface and main screen. (a) Main menu, (b) search result pages, (c) type of input sequence, (d) direct input of a sequence, (e) sequence upload from a file, (f) sequence position within the query sequence, (g) adding extra 5′ and 3′ sequences from the query, (h) genome assembly and their identity percentage, (i) Target locus default and user defined values.

The screenshot of the input interface for UniPrimer is shown on Figure 2. The main menu is located on the top right corner of the figure (Figure 2a) where the user can find information about tools, contacts, and a tutorial. The topmost tabs (Figure 2b) in the main screen are pages that will contain search results for each step after a query sequence is submitted. After selecting the type of query sequence and its assembly (Figure 2c), the user pastes the target sequence directly into text area (Figure 2d) or uploads it from a file (Figure 2e). The position (Figure 2f) option can be used if the user wants to limit the query to a specific chromosome or region. In contrast, it is also possible to add extra sequences at the 5′ and 3′ ends of the query sequence (Figure 2g). Next go the genome types, their assembly versions, and identity percentages (Figure 2h) where up to six-genome assembly similarities are searched at once. The last option in the input form is selecting the target locus (Figure 2i) in which user can select either default values (+500, −500) or define the positions that the PCR product should contain.

#### 3.1.2. BLAT Search

**Figure 3:**
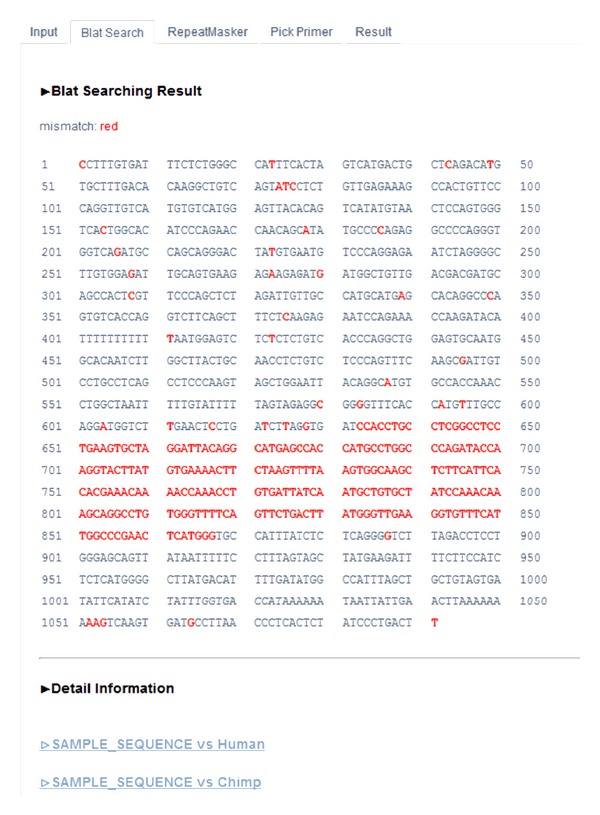
BLAT search results. Matching and mismatching bases are in black and red, respectively.

BLAT step allows a user to find orthologous between a query sequence and several other genomes. A BLAT search result is shown in Figure 3. Identical bases between the query sequence and its corresponding sequences from other primates are colored black but mismatches between sequences are all combined and marked as red letters. However, below it, user can find links to a detailed comparison of a query sequence with each of selected genomes, separately.

It is possible to design primers on mismatching areas, but 100% conserved sequence among multiple species gives the best PCR result. Notably, UniPrimer's BLAT search compares the sequences from more than two species at the same time. As such, this approach is particularly helpful for users conducting PCR assay with more than two species.

#### 3.1.3. RepeatMasker

**Figure 4:**
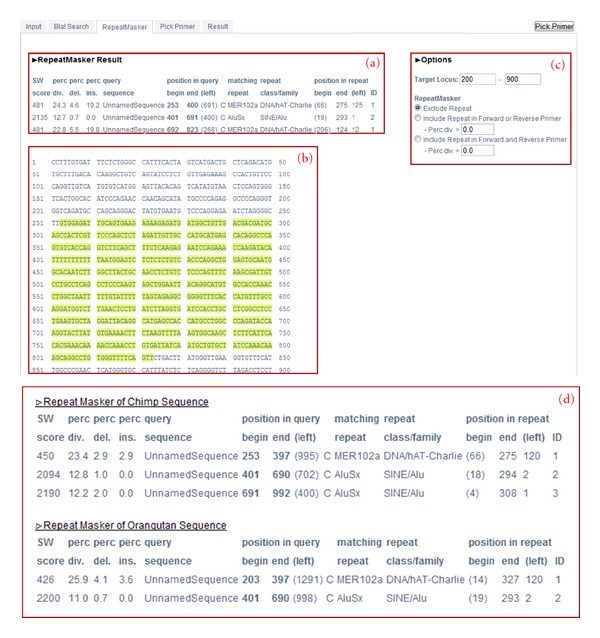
RepeatMasker search result. (a) Detailed annotation of repeats in a table format. (b) Sequence format with masked repeats; masked areas are highlighted in light green. (c) Extra options for selecting the target locus and primer type. (d) Separate tables summarize repetitive elements found in each of selected primate genomes (orthologous region).

RepeatMasker helps to eliminate the building of any candidate primer containing repetitive elements by masking them. Primers that are built on unmasked areas are more successful than those built on masked areas. The output result of RepeatMasker (Figure 4) returns a detailed annotation of repeat elements that are present in the query sequence. The annotation is shown in two formats; a table format contains a list of the repeats (Figure 4a), and repeat sequences are highlighted with light green color in the other format (Figure 4b). On the right side of the window, the user can find options to change the target locus and the type of primer (Figure 4c). After selecting the options, the user obtains a list of designed primers by clicking “Pick primer” on the top right corner of the main screen (step C-3 in Figure 1). Under the masked sequence, there are separate tables that describe masked areas of genomes, separately.

Details about the table and options are listed below.

*How to read the table result* in Figure 4a:

This section shows the output of RepeatMasker (http://www.repeatmasker.org/cgi-bin/WEBRepeatMasker/).

SW score: Smith-Waterman score of the match.
Perc. div.: percentage of substitutions in the matching region compared to the consensus.
Perc. del.: percentage of bases opposite a gap in the query sequence (deleted bp).
Perc. ins.: percentage of bases opposite a gap in the repeat consensus (inserted bp).
Query sequence: name of input sequence.
Position in query: beginning and ending are starting and ending positions for the match in the query sequence respectively. (left) indicates the number of bases in query sequence past the end position of the match.
Matching repeat: name of the matching interspersed repeat.
Repeat class/family: the class of the repeat.
Position in repeat: beginning and end are starting and ending positions for the match in the database sequence. (left) indicates the number of bases in the repeat consensus sequence prior to beginning the match.

*How to adjust options *in Figure 4c:

Target locus: the minimum region of PCR amplification.
Exclude repeat: avoid designing primers for repeat sequences.
Include repeat in forward primer or reverse primer: one could include repeat sequences for either forward or reverse primers.
Include repeat in forward primer and reverse primer: could include repeat sequences for both the forward and reverse primers. Additionally, the user can set the minimum perc div. of repetitive elements that belong to the primer. Higher perc div. leads to better results.

#### 3.1.4. Pick Primer

**Figure 5:**
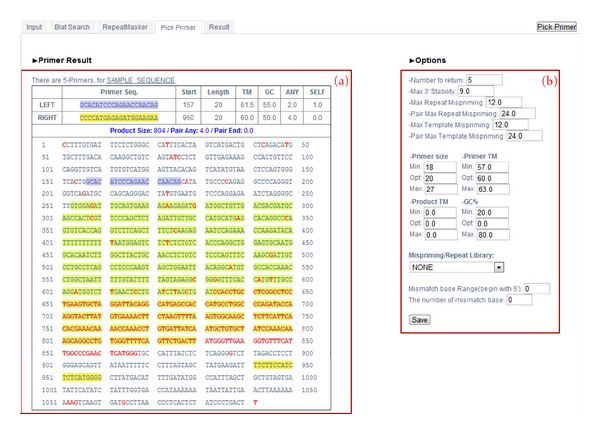
Pick primer result. (a) Table showing primers, their detailed information, and position in the sequence. (b) Additional options for the Pick primer step.

Pick primer is linked to Primer3Plus, which selects the primer pairs that fit best to the selected parameters and orders them by quality. The Pick primer output is shown in Figure 5. The left side table (Figure 5a) will contain detailed information of all possible primers present in the query sequence, as well as the whole sequence with BLAT and RepeatMasker output results shown together; the left primers are highlighted in purple, the right primers are highlighted in yellow, the BLAT mismatches are marked in red, and the masked repeats are highlighted in light green. 

Similar to the RepeatMasker step, if the user wants more specified primers he can recalculate the Pick primer step after customizing with additional options (Figure 5b) on the right-hand side of the page.

*How to read a table result *(Figure 5a):

Primer seq.: LEFT and RIGHT primers are highlighted in purple and yellow colors, respectively.
Start: the position of the 5′ base of the primer. For left primers it is the position of the leftmost base and for right primers it is the rightmost base.
Length: length of the primer.
TM: melting temperature of the primer.
GC: the percent of G and C bases in the primer.
ANY: self-complementary score of the primer.
SELF: 3′ self-complementary of the primer.

*How to adjust options for Pick primer *(Figure 5b):

This option is followed by Primer3 [11].

Number to return: the maximum number of primer pairs to return. Setting this parameter to a large value will increase running time.
Max 3′ stability: the maximum stability for the last five 3′ bases of a left or right primer. Larger numbers mean more stable 3′ ends.
Max Repeat Mispriming: the maximum allowed weighted similarity with any sequence in the Mispriming Library. Default is 12.
Pair Max Repeat Mispriming: the maximum allowed sum of similarities of a primer pair (one similarity for each primer) with any single sequence in the Mispriming Library.
Max Template Mispriming: the maximum allowed similarity to ectopic sites in the sequence from which you are designing the primers.
Pair Max Template Mispriming: the maximum allowed summed similarity of both primers to ectopic sites in the sequence from which the primers are designed.
Primer Size: minimum, optimum, and maximum lengths of the primer oligo.
Primer TM: minimum, optimum, and maximum melting temperatures for the primer oligo in Celsius.
Product TM: minimum, optimum, and maximum melting temperatures for the amplicon.
GC %: minimum, optimum, and maximum percentages of Gs and Cs in any primer.
Mispriming/Repeat library indicates what mispriming library (if any) should be used to screen for interspersed repeats or for other sequence to avoid as a primer location.

We added a mismatch base range (begin with 5′) and the number of mismatch bases options. A mismatched base at the 5′ end of the primer is more tolerable than that at the 3′ end to obtain a successful PCR amplification.

Mismatch base Range (begin with 5′ end): it allows for a mismatched base from the 5′ end.

The number of mismatch bases: it allows the number of mismatched base within the mismatched base range.

#### 3.1.5. Final Result

**Figure 6:**
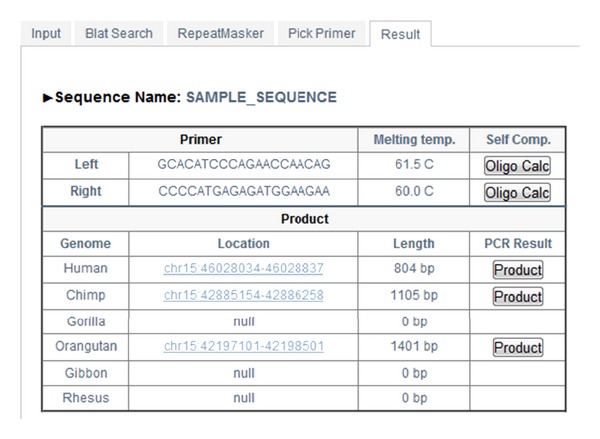
Final primer result.

**Figure 7:**
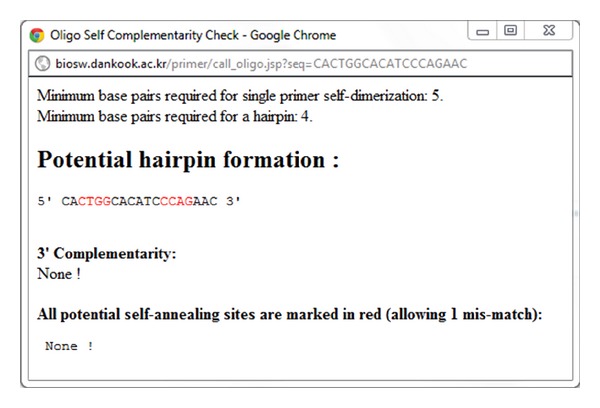
Oligo self-complementary check window.

**Figure 8:**
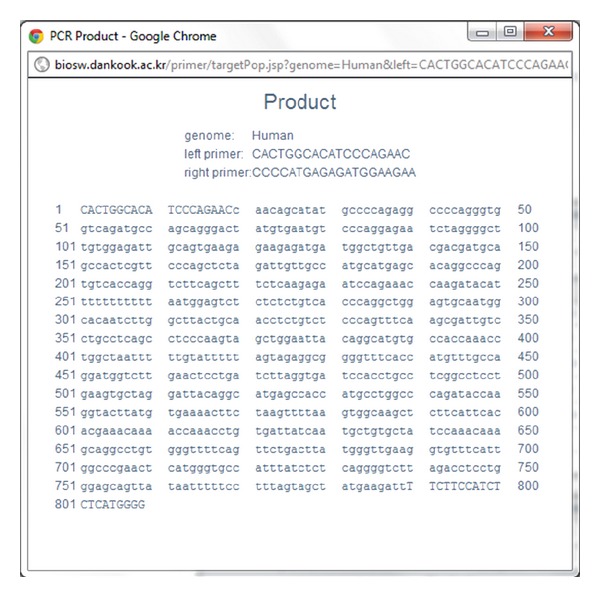
Polymerase chain reaction (PCR) product window.

The last page of UniPrimer contains the primer information picked by the user (Figure 6). The upper part of the table shows the left and right primer sequences and their melting temperatures in Celsius.  Figure 7 shows a pop-up window that the user can reach by clicking the “OligoClac” button on the Figure 6 and potential hairpin, 3′ complementarity, and self-annealing sites of candidate primers are calculated and displayed. The lower part of the table in Figure 6 shows the genome type, the genomic position, and the expected size of the PCR product. Nationally, the user can access UCSC *In-Silico* PCR by clicking the “Product” button on the right side of Figure 6 (Figure 8). In the results of *In-Silico* PCR, primers that are written in capital letters and sequences that lie between them are written in small letters (Figure 8).

## 4. Performance Comparison

As mentioned before, UniPrimer is constructed to compare up to six primate sequences at once and design primers on the conserved regions among them. We believe that using this tool to design PCR primers would save great deal of time and reduce possible errors because of two reasons described below. First, UniPrimer is not required to submit the sequence over and over again to match it with each of selected genomes. Second, since masked and mismatching areas in the query sequence are marked accordingly on Pick primer step (Figure 5), user is able to determine a proper region for designing good primers.

To estimate the performance and scalability, we used UniPrimer to design PCR primers using a sample sequence that is related to *Alu* recombination-mediated deletions in the human genome [8] as a query. In addition, we designed primers for the same sequence using a manual method to test the efficiency of UniPrimer.  Table 2 shows the approximate time consumed by UniPrimer and the manual method to design the primers for the sample sequence. The result indicates that UniPrimer works much faster than that of the manual method. The user needs to open each web-based tool separately for the manual method and submit a query sequence on each window. The loading time required to return a result depends on the length of the query sequence and the number of genomes that are selected by user. Any error that occurs will add time. 

## 5. Conclusions

We developed UniPrimer, a web-based tool to design PCR- and DNA-sequencing primers. This tool is able to find conserved regions across different primate species and designs primers for the region. Then, users are allowed to select various options for picking the best primer for their purpose. UniPrimer was developed to reduce the time required for designing PCR primers and the errors that occur during the process. We conducted a performance test to determine whether this tool works as intended, and the result showed that UniPrimer was easy to use and saved time and effort. In conclusion, we believe that UniPrimer could be a useful tool for comparative analyses of primate genomes.

## Figures and Tables

**Table 1:** UniPrimer development environment.

Items	Tools
Script language	Java server pages (JSP)
User interface	HTML, jQuery
Servlet container	Apache tomcat 6.0
Browser	Chrome, Mozilla, Safari

**Table 2:** Comparison of time consumed with UniPrimer and manual work measured in minutes.

UniPrimer	Manual work
2.5 minutes	8 minutes
